# ATR Deficiency Impairs DNA Damage Repair and Accelerates Cellular Senescence in Bovine Mammary Epithelial Cells, Leading to Lactation Dysfunction

**DOI:** 10.3390/ani15101419

**Published:** 2025-05-14

**Authors:** Qijun Zhou, Zijian Geng, Shuai Lian, Jianfa Wang, Rui Wu

**Affiliations:** 1 College of Animal Science and Veterinary Medicine, Heilongjiang Bayi Agricultural University, No. 5 Xinfeng Road, Daqing 163319, China; 2 China Key Laboratory of Bovine Disease Control in Northeast China, Ministry of Agriculture and Rural Affairs, Daqing 163319, China; 3 College of Biology and Agriculture, Jiamusi University, Jiamusi 154007, China

**Keywords:** production lifespan, ATR, bovine mammary health, DDR

## Abstract

Mammary glands in cows are highly dynamic, making genomic stability particularly crucial. Continuous lactation and self-renewal of these glands are primary contributors to genomic instability. **Results:** We employed transcriptomic and proteomic methods to analyze the expressional characteristics in the mammary glands of cows with varying levels of milk production. Our findings indicated differences in relevant pathways, including DNA damage repair and apoptosis, which are influenced by increasing parity. Notably, ATR protein levels in the mammary glands of low-yield dairy cows were reduced. Following in vitro silencing of ATR, β-galactosidase content increased in aging mammary epithelial cells, with cell cycle arrest in the G2 and S phases. Secretory phenotypes associated with aging, including IL-6, IL-10, IL-1β, INF-γ, and IL-2, were elevated, along with increased TNF-α content. The expressions of DNA repair-related proteins, including PIG3, PARP1, and Cleaved caspase3, were upregulated, and SP1 expression was decreased. Furthermore, the expressions of cytochrome C and BAK increased, and ATR silencing inhibited mTOR and STAT5 lactation signaling pathways, resulting in elevated STAT3 protein levels associated with mammary gland degeneration. **Conclusions:** This study emphasizes the significance of the ATR protein in the mammary glands of dairy cows, contributing valuable insights into maintaining their health and presenting a novel perspective on strategies to enhance their lifespan.

## Simple Summary

As parity increases, the health of dairy cows’ mammary glands declines, leading to reduced milk yield and a shorter productive lifespan. Variation in mammary gland health is the primary cause of differences in milk production. Therefore, studying the differences in mammary gland health among dairy cows is crucial for promoting healthy livestock farming. Our study demonstrates that with increasing parity, differences in mammary gland health manifest in signaling pathways related to immune and inflammatory responses, as well as DNA damage repair. Alterations in ATR and PIG3, which affect DNA damage repair in mammary gland cells, contribute to the polarized milk yield. A reduction in ATR results in cell cycle arrest and apoptosis in bovine mammary epithelial cells (BMECs), an increase in senescence-associated secretory phenotype (SASP) content, decreased mTOR expression, elevated STAT3 protein levels, and significantly reduced mRNA levels of CSN2 and CSN3.

## 1. Introduction

The mammary glands of cows are crucial biological and economic organs, functioning as the sole site for milk synthesis and secretion. Increased milk production is associated with enhanced growth and metabolism in cows; however, this elevates the risk of infections, toxic pressure, and substantial DNA damage [1]. Conversely, the lactation period necessitates the maintenance of cell numbers, cellular activity, and synthesis of various nutrients that are essential for lactation. Consequently, the replication pressure on the breast cell genome intensifies [2,3]. In animal husbandry, the aim is to obtain high-yield, long-lived dairy cows. While such cows exist in practice, it remains unclear whether specific genes within their breast tissue confer resistance to DNA damage.

Ataxia telangiectasia and Rad3-related protein (ATR), a DNA damage sensor, activates cell cycle checkpoint signals [4]. It promotes the phosphorylation of various downstream target proteins involved in inhibiting DNA replication and mitosis [5,6]. ATR facilitates DNA repair, recombination, and apoptosis [7], which are crucial processes for maintaining the stability and integrity of the genome during the remodeling of breast tissue [8].

Previous research on improving milk production in lactating cows has mainly focused on the diagnosis and prognosis of breast diseases, along with the exploration of milk production-related traits. How does milk production of cows passively decrease during the lactation cycle? What are the underlying reasons for the aging differences observed in the mammary glands of dairy cows during the lactation cycle? The changes in the lactation function of breast cells during the aging process with increasing parity have not been explored.

## 2. Materials and Methods

### 2.1. Experimental Animals and Grouping

The study was approved and supported by the Animal Welfare Ethics Committee of Heilongjiang Bayi Agricultural University (approval no: DWKJXY2024057). Eighteen Holstein cows from a large-scale ranch in Daqing City, Heilongjiang Province, China, were chosen as experimental subjects based on their health, similar physique, and peak lactation period (21–100 days). The cows were categorized into six groups according to their milk production and parity: high-yield groups (daily lactation volume > 35 kg/day) comprising parity 1 (HP1), parity 5 (HP5), and parity 6 (HP6), and low-yield groups (20 kg/day ≤ daily lactation volume ≤ 30 kg/day) comprising parity 1 (LP1), parity 5 (LP5), and parity 6 (LP6), with three cows in each group. Milk separation cells were obtained for transcriptome and proteome sequencing, and differences in protein expression were analyzed. Subsequently, primary breast epithelial cells were cultured in vitro, and selected ATR proteins were silenced using siRNA. Relevant changes in breast epithelial cells following ATR silencing were investigated using flow cytometry, qPCR, and Western blot analyses.

### 2.2. Preparation and Detection of Samples for Transcriptomics and Proteomics Analyses

The breasts were wiped with 75% alcohol for disinfection. The first five handfuls of milk were discarded, and the remaining milk was collected in a centrifuge bottle (3120–0250, Thermo Fisher, Waltham, MA, USA). The sample was centrifuged at 1000× *g* for 20 min using a CR21GIII centrifuge (Hitachi, Tokyo, Japan). After centrifugation, the cream and supernatant were carefully removed. Next, 35 mL of 1× PBS (P1010, Solarbio, Beijing, China) was added to the pellet, the cells were resuspended in a 50 mL centrifuge tube and centrifuged again, and the supernatant was discarded. The process was repeated until a clear supernatant was obtained. The supernatant was discarded, 2 mL of 1× PBS was added to the pellet and resuspended, and the solution was transferred to two low-adsorption 1.5 mL EP tubes. The sample was centrifuged at 1000× *g* for 10 min, the supernatant was discarded, and the pellet was frozen in liquid nitrogen for 15 min. Finally, the samples were stored at −80 °C for sequencing by Novogene Co., Ltd., Beijing, China.

### 2.3. Primary Bovine Mammary Cell (BMEC) Culture

Primary BMECs were provided by the Provincial Key Laboratory of Cattle Disease Prevention and Control (Heilongjiang Bayi Agricultural University, Daqing, Heilongjiang, China) and cultured with DMEM/F12 (#SH30023.01, HyClone, Logan, UT, USA) containing 10% fetal bovine serum (#SH30084.03, HyClone, Logan, UT, USA) and 1% streptomycin mixture (#P1400, Solarbio, Beijing, China). The solution was changed daily. The cells were allowed to grow until they reached approximately 80% confluency and were digested and passaged with 0.25% trypsin (#T1320, Solarbio, Beijing, China). All cells were cultured at 37 °C in an incubator with 5% CO_2_ (MCO-230AICUVL-PC, PHCBI, Loughborough, UK).

### 2.4. ATR Knockdown in BMECs

siRNA constructs targeting ATR were synthesized by HANBIO Technology (Shanghai, China). The sequence of the sense strand was 5′-GAUAGGUUGUUCUGCUATT-3′, and that of the corresponding antisense strand was 5′-UAGCAGAACACACCUAUCTT-3′. Specificity for the bovine genome was confirmed through a BLAST (version 2.14.0) search, and random sequences with a similar GC content to the bovine genome were utilized as negative controls. Random sequences were as follows: sense strand, 5′-UUCUCCGAACGUGUCACGUTT-3′, and antisense strand, 5′-ACGUGACACGUUCGGAGAATT-3′. Transfection was performed using Lipofectamine™ 3000 (#L3000015, Thermo Fisher Scientific, Waltham, MA, USA) following the manufacturer’s instructions. Specifically, 20 μM of ATR siRNA and 20 μM of NC siRNA were added to a serum-free medium containing Opti-MEM™ (#31985070, Thermo Fisher Scientific, Waltham, MA, USA), and transfected cells were incubated for 6 h. Next, the medium was replaced with one containing 10% FBS, and the cells were collected after 48 h. The transfection efficiency was assessed, and subsequent experiments were conducted using both the fluid and the cells.

### 2.5. RNA Extraction, cDNA Synthesis, and Quantitative PCR (qPCR)

Total RNA was extracted from BMECs using TRIzol (#R1100, Solarbio, Beijing, China) following the manufacturer’s instructions. The RNA was reverse transcribed into the corresponding cDNA using the PrimeScript™ RT Reagent Kit with gDNA Eraser (Perfect Real Time) (#RR047A, TAKARA, Shiga, Japan). qPCR was performed using the CFX 96 Real-Time System (Bio-Rad, Hercules, CA, USA). Gene expression was calculated using the 2^−ΔΔCT^ method, with β-actin as the reference gene, and the analysis was conducted using Microsoft Excel (2021 version). The specific gene sequences are listed in Table 1.

### 2.6. Protein Extraction and Western Blotting

The cell culture medium was discarded, and RIPA lysis (#P0013 B, Beyotime, Haimen, China) was added to the cells. The protein concentration was determined using the BCA protein concentration assay kit (#P0010, Beyotime, Haimen, China). Proteins were separated by SDS-PAGE before being transferred onto a PVDF membrane (#IPVHO0010, Millipore, Billerica, MA, USA). After blocking with a protein-free rapid blocking solution (#G2052, Servicebio, Wuhan, China), the membranes were incubated with primary antibodies overnight at 4 °C. The signal was detected using the hypersensitive ECL chemiluminescence kit (#P10100, NCMBiotech, Suzhou, China), and imaging was performed with the Amersham Imager 600 (GE Healthcare, Chicago, IL, USA). Each electrophoresis lane represents an independent experiment with three replicates. The results were analyzed for optical density using ImageJ 1.54f, with β-actin as an internal reference. Then, *t*-test and plotting were conducted using GraphPad 9.5.

Antibodies against ATR (1:1000, #AF4767), PIG3 (1:1000, #AF9159), and p-JAK2 (1:1000, #AF3022) were purchased from Affinity Biosciences (Jiangsu, China). Those for SP1 (1:2000, #21962-1-Ap), Cytochrome C (1:5000, #66264-1-Ig), STAT3 (1:2000, #10253-2-AP), and Cleaved caspase3 (1:2000, #325128-1-AP) were obtained from Proteintech (Wuhan, China). Antibodies against BAK1 (1:2000, #bs-1284R), AKT (1:2000, #bs-6951R), p-AKT (1:2000, #bs-0876R), and JAK2 (1:2000, #bs-23003R) were sourced from Bioss (Beijing, China). Antibodies against p-STAT3 (1:2000, #9145), p-STAT5 (1:2000, #9359), and STAT5 (1:2000, #94205) were procured from CST (Danvers, MA, USA). The antibody against mTOR (0.1–1 μg/mL, #ab83495) was acquired from Abcam (Cambridge, UK), and those against RARP1 (1:2000, #A0942) and β-actin (1:80,000, #AC026) were purchased from ABclonal (Wuhan, China). Secondary antibodies included horseradish peroxidase-labeled goat anti-mouse IgG (1:8000, #SA00001-1) and goat anti-rabbit IgG (1:8000, #SA00001-2), both procured from Proteintech (Wuhan, China).

### 2.7. Flow Cytometric Analysis of Cell Cycle

The Cell Cycle and Apoptosis Detection Kit (#C1052, Beyotime, Haimen, China) was used following the manufacturer’s instructions. The cell culture medium was collected, cells were trypsinized (#C0201, Beyotime, Haimen, China) for 2 min at room temperature, and the cell culture medium was added to stop digestion. Cells were collected and centrifuged at 1000× *g* for 3 min. The supernatant was discarded, and pre-cooled PBS was added to wash the pellet. The resuspended pellet was centrifuged, and the supernatant was discarded. Next, 1 mL of pre-cooled 70% ethanol was added, and cells were resuspended in a 1.5 mL EP tube at 4 °C for 24 h. The supernatant was discarded by centrifugation, and the pellet was washed with pre-cooled PBS. The supernatant was discarded by centrifugation, and 500 μL of Protariod iodide staining solution was added. Cells were incubated at 37 °C for 30 min, collected, and subjected to flow cytometry on the CytoFLEX (Beckman Coulter, Pasadena, CA, USA) platform. The CytExpert (version 2.4) and FlowJo (version V10.8.1) software were used to collect and analyze all data.

### 2.8. Detection of Senescent Cells by β-Galactosidase

Cell senescence was detected by β-galactosidase staining following the manufacturer’s instructions as follows: The cell culture medium was discarded, and 1 mL of fixation solution was added to fix the cells for 15 min at 20 °C. After fixation, the fixation solution was removed and the cells were washed with PBS. The PBS was discarded and 1 mL of staining solution was added. The cells were incubated overnight at 37 °C and imaged under a microscope (MI52-N, Mshot, Guangzhou, China).

### 2.9. Detection of Secretory Phenotypes Related to Aging

Following the manufacturer’s instructions, the corresponding ELISA kits were utilized to detect bovine-derived IL-4 (#SEA077Bo, Cloud-Clone Corp, Houston, TX, USA), IL-1β (#SEA563Bo, Cloud-Clone Corp), IFN-γ (#SEA049Bo, Cloud-Clone Corp), IL-10 (#SEA056Bo, Cloud-Clone Corp), IL-2 (#SEA073Bo, Cloud-Clone Corp), IL-6 (#SEA079Bo, Cloud-Clone Corp), IL-8 (#SEA080Bo, Cloud-Clone Corp), and TNF-α (#SEA133Bo, Cloud-Clone Corp) levels in the cell supernatant.

### 2.10. Statistical Analysis

Each experiment included a minimum of three biological replicates, with data expressed as mean ± standard error. The transcriptomic raw data were processed using bioinformatics tools, and differentially expressed genes (DEGs) were analyzed with DESeq2 (version 1.20.0). The *p*-values were adjusted using a negative binomial distribution model and the Benjamini–Hochberg method. In this study, DEGs were filtered based on the following criteria: *p*-value ≤ 0.05 and |log2FoldChange| ≥ 1.0. Proteomic analysis was conducted using DIA-NN (version 1.9) software (Direct DIA) to generate solution profiles from the original data and search species libraries. Retention time correction was performed using the iRT standard added to the samples, with a precursor ion Q-value cutoff set at 0.01. Subsequently, the protein quantification results were statistically analyzed using a *t*-test, and those with a difference ratio (fold change, FC) greater than 1.2 were considered differentially expressed proteins (DEPs). Additionally, identified proteins were analyzed using the InterProScan (version 103.0) software. Student’s *t*-test was employed for intergroup comparisons, and data were analyzed using GraphPad Prism 10, with statistical significance defined at *p* < 0.05.

## 3. Results

### 3.1. Multiomics Analysis and Verification

**Figure 1:**
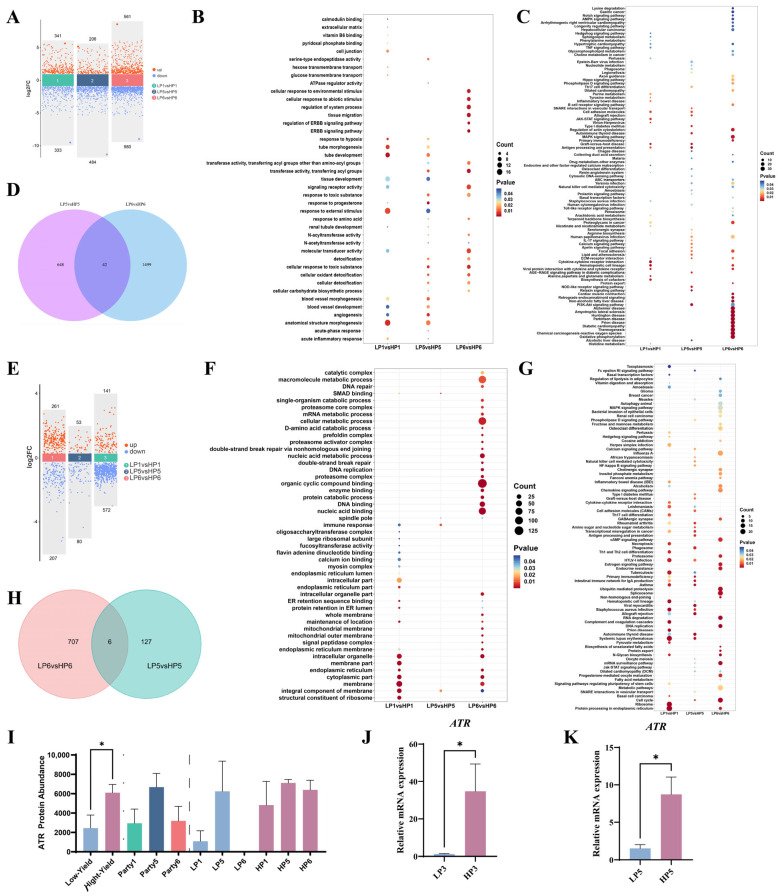
Omics differences in mammary tissues of high- and low-yielding dairy cows at different lactation periods. (**A**) Volcano plot of differentially expressed genes (DEGs). (**B**) GO enrichment analysis for DEGs. (**C**) KEGG enrichment analysis for DEGs. (**D**) Venn diagram for DEGs. (**E**) Volcano plot of differentially expressed proteins (DEPs). (**F**) GO enrichment analysis for DEPs. (**G**) KEGG enrichment analysis for DEPs. (**H**) Venn diagram for DEPs. (**I**) Proteomics results for ATR protein. (**J**,**K**) qPCR validation of proteomics data. Significant differences are indicated as * *p* < 0.05. Data are presented as mean ± SEM. In the volcano plots, the x-axis represents different comparison groups, and the *y*-axis represents the fold change in DEG (log2FC value ≥ 1.0) and DEP (log2FC value ≥ 1.2) expression.

We first conducted a comparative transcriptomic analysis of dairy cows with varying milk yields to explore the differences in the transcriptional levels of mammary tissues between high- and low-yielding dairy cows. Figure 1A illustrates the DEGs between the groups. A total of 2905 DEGs were identified among the first, fifth, and sixth lactations between high- and low-yielding dairy cows. Subsequently, GO enrichment analysis was performed for the DEGs. As shown in Figure 1B, the DEGs between high- and low-yielding cows in the first, fifth, and sixth lactations were significantly enriched in pathways, including tube development, anatomical structure morphogenesis, tissue development, and response to external stimulus. KEGG enrichment analysis was conducted for DEGs, and as shown in Figure 1C, the DEGs across the first, fifth, and sixth lactations were commonly enriched in pathways with high abundance, including the PI3K–Akt signaling pathway, oxidative phosphorylation, histidine metabolism, of regulation the actin cytoskeleton, and cytokine–cytokine receptor interaction. A Venn diagram was constructed (Figure 1D) to identify the genes that accumulate differences between high- and low-yielding dairy cows across lactations. Forty-two DEGs were identified (Supplementary Table S1). Among them, *IL-6*, related to the senescence-associated secretory phenotype; *TP53I3* (*PIG3*), involved in DNA damage repair; and *LALBA*, associated with milk quality, were differentially expressed.

We subsequently conducted a proteomic analysis on dairy cows with varying milk yields to investigate the differences in protein levels of mammary tissues between high- and low-yielding dairy cows. Figure 1E presents the results of the comparison of DEPs showing significant differences (*p* < 0.05). A total of 468 DEPs were identified between LP1 and HP1, of which 261 were upregulated and 207 were downregulated; 133 DEPs were identified between LP5 and HP5, with 53 upregulated and 80 downregulated; and 713 DEPs were identified between LP6 and HP6, of which 141 were upregulated and 572 were downregulated. As shown in Figure 1F, DEPs were enriched in GO terms, including DNA repair, nucleic acid metabolic process, double-strand break repair, DNA replication, enzyme binding, DNA binding, nucleic acid binding, and immune response. Figure 1G illustrates that the DEPs were enriched in KEGG pathways related to the organic substance metabolic process, cellular metabolic process, ATP binding, protein metabolic process, organic cyclic compound binding, kinase activity, and serine-type endopeptidase activity. Figure 1H shows DEPs between high- and low-yielding dairy cows across lactations. The DNA repair protein, ATR, differed significantly between high- and low-yielding cows, and its expression was not detected in the low-yielding cows at the sixth lactation (Figure 1I). ATR is central to DNA damage repair. Combining the results of the transcriptomic analysis, we speculate that the decrease in ATR expression in the mammary tissues of cows may have been due to the increase in lactation number. To further validate the results of -omics analysis, healthy high-yielding cows (milk yield > 35 kg/day) and low-yielding cows (20 < milk yield ≤ 30 kg/day) in their third and fifth lactations were tested. As shown in Figure 1J,K, compared with high-yielding cows, the mRNA level of *ATR* in the mammary tissues of low-yielding cows was significantly lower (*p* < 0.05).

### 3.2. Verifying ATR Knockdown Efficiency in Primary BMECs

**Figure 2:**
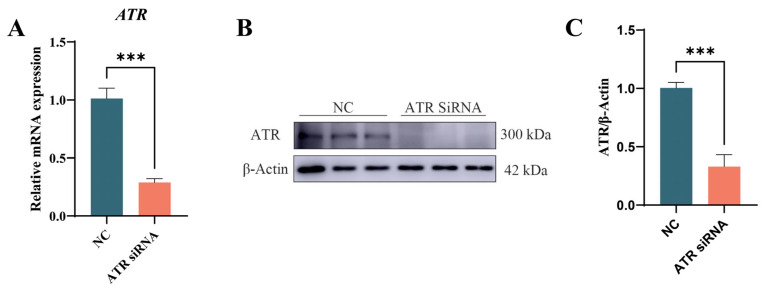
Transfection of ATR siRNA constructs. (**A**) qPCR validation at 48 h post-transfection. (**B**) Protein levels at 48 h post-transfection. (**C**) Grayscale analysis of the ATR protein level. Significant differences are indicated as *** *p* < 0.05.

ATR was knocked down in primary BMECs to investigate its effects. After 48 h of transfection with ATR siRNA, compared with the negative control, both the mRNA (Figure 2A) and the protein (Figure 2B,C) levels were significantly reduced (*p* < 0.001).

### 3.3. Effects of ATR Silencing on Senescence-Associated β-Galactosidase Expression and Cell Cycle in BMECs

**Figure 3:**
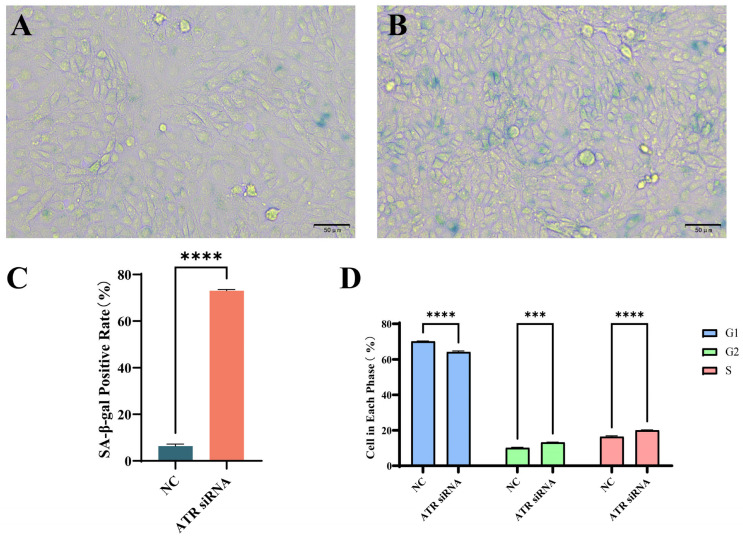
Effects of ATR silencing on senescence-associated β-galactosidase content and cell cycle in BMECs. (**A**) Negative control group. (**B**) ATR-silenced group. (**C**) The activity level of senescent galactosidase in BMECs via Image J analysis. (**D**) Analysis of cell cycle changes between the NC group and the ATR siRNA group. Significant differences are indicated as *** *p* < 0.001, **** *p* < 0.0001. Scale bar = 50 μm.

Cell cycle arrest and increased levels of senescence-associated β-galactosidase are significant hallmarks of cellular senescence. Compared with the negative control (Figure 3A,C), the level of senescence-associated β-galactosidase in BMECs increased significantly after 48 h of ATR silencing (Figure 3B,C). Cell cycle analysis showed that after 48 h of ATR silencing, the G1 phase was shortened (*p* < 0.0001), while the G2 phase (*p* < 0.001) and S phase (*p* < 0.0001) were prolonged in BMECs (Figure 3D).

### 3.4. Effects of ATR Silencing on Senescence-Associated Secretory Phenotype (SASP) in BMECs

**Figure 4:**
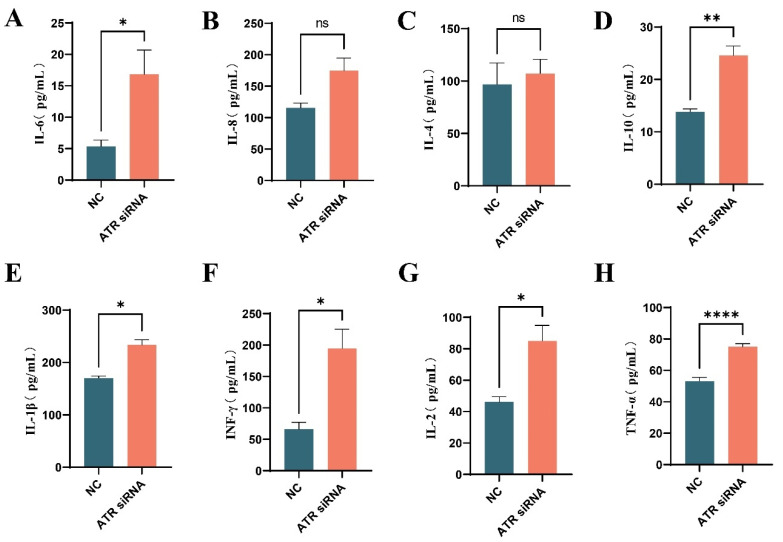
Effects of ATR silencing on SASP. (**A**) IL-6 levels in BMEC culture supernatant. (**B**) IL-8 levels in BMEC culture supernatant. (**C**) IL-4 levels in BMEC culture supernatant. (**D**) IL-10 levels in BMEC culture supernatant. (**E**) IL-1β levels in BMEC culture supernatant. (**F**) INF-γ levels in BMEC culture supernatant. (**G**) IL-2 levels in BMEC culture supernatant. (**H**) TNF-α levels in BMEC culture supernatant. Significant differences are indicated as * *p* < 0.05, ** *p* < 0.01, **** *p* < 0.0001, ns *p* > 0.05.

As shown in Figure 4, the levels of IL-6 (*p* < 0.05), IL-10 (*p* < 0.001), IL-1β (*p* < 0.05), INF-γ (*p* < 0.05), IL-2 (*p* < 0.05), and TNF-α (*p* < 0.0001) in the culture supernatant of BMECs increased significantly after ATR silencing. Although the levels of IL-8 and IL-4 increased, the differences were not statistically significant (*p* > 0.05).

### 3.5. Effects of ATR Silencing on DNA Damage Repair and Apoptosis in BMECs

**Figure 5:**
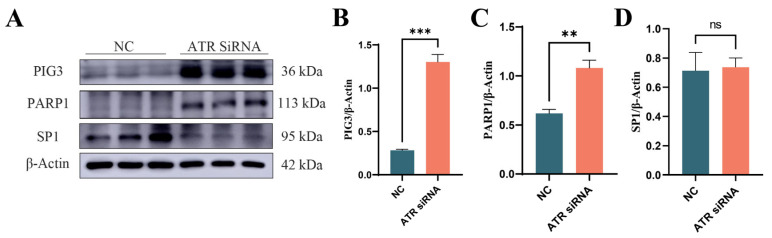
Effects of ATR silencing on DNA damage repair. (**A**) Western blot image. (**B**) Grayscale analysis of PIG3 protein. (**C**) Grayscale analysis of PARP1 protein. (**D**) Grayscale analysis of SP1 protein. Significant differences are indicated as ** *p* < 0.01, *** *p* < 0.001, ns *p* > 0.05.

To assess the impact of ATR silencing on DNA damage repair in cells, the expression levels of PIG3, PARP1, and SP1 were examined. As shown in Figure 5, the expressions of PIG3 (*p* < 0.001) and PARP1 (*p* < 0.01) were significantly increased, while there was no significant effect on the expression of SP1 (*p* > 0.05).

**Figure 6:**
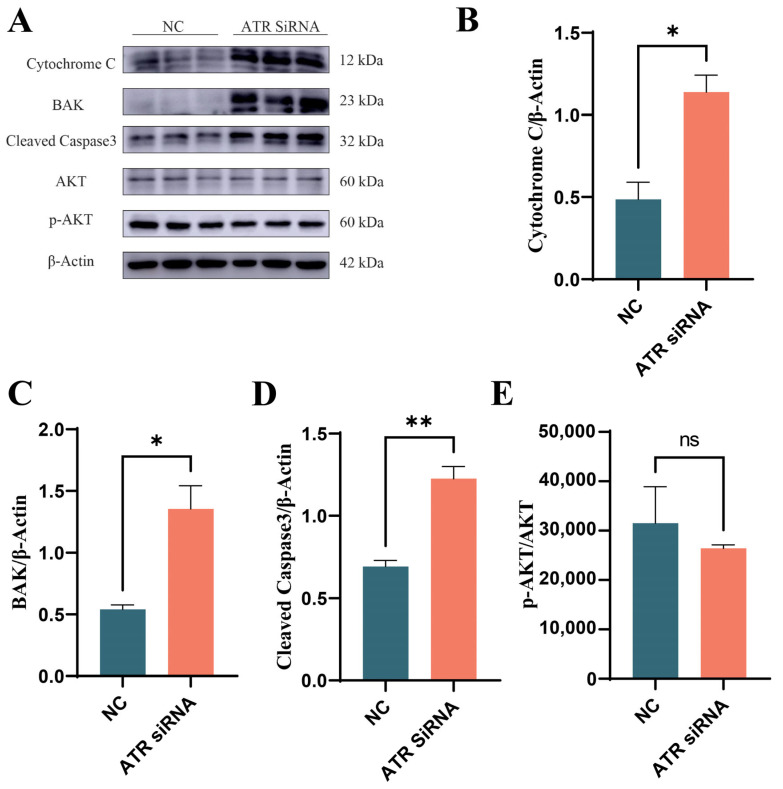
Effects of ATR silencing on apoptosis. (**A**) Western blot image. (**B**) Grayscale analysis of cytochrome C protein. (**C**) Grayscale analysis of BAK1 protein. (**D**) Grayscale analysis of Cleaved caspase 3 protein. (**E**) Grayscale analysis of AKT/p-AKT protein. Significant differences are indicated as * *p* < 0.05, ** *p* < 0.01, ns *p* > 0.05.

To further determine the impact of ATR silencing on apoptosis, the expression levels of cytochrome C, BAK1, Cleaved caspase3, and p-AKT/AKT were assessed. As shown in Figure 6, the levels of cytochrome C (*p* < 0.05), BAK1 (*p* < 0.05), and Cleaved caspase3 (*p* < 0.01) were significantly upregulated, while the expression ratio of p-AKT/AKT decreased, although the difference was not statistically significant (*p* > 0.05).

### 3.6. Effect of ATR Silencing on Lactation-Related Signaling Pathways in BMECs

**Figure 7:**
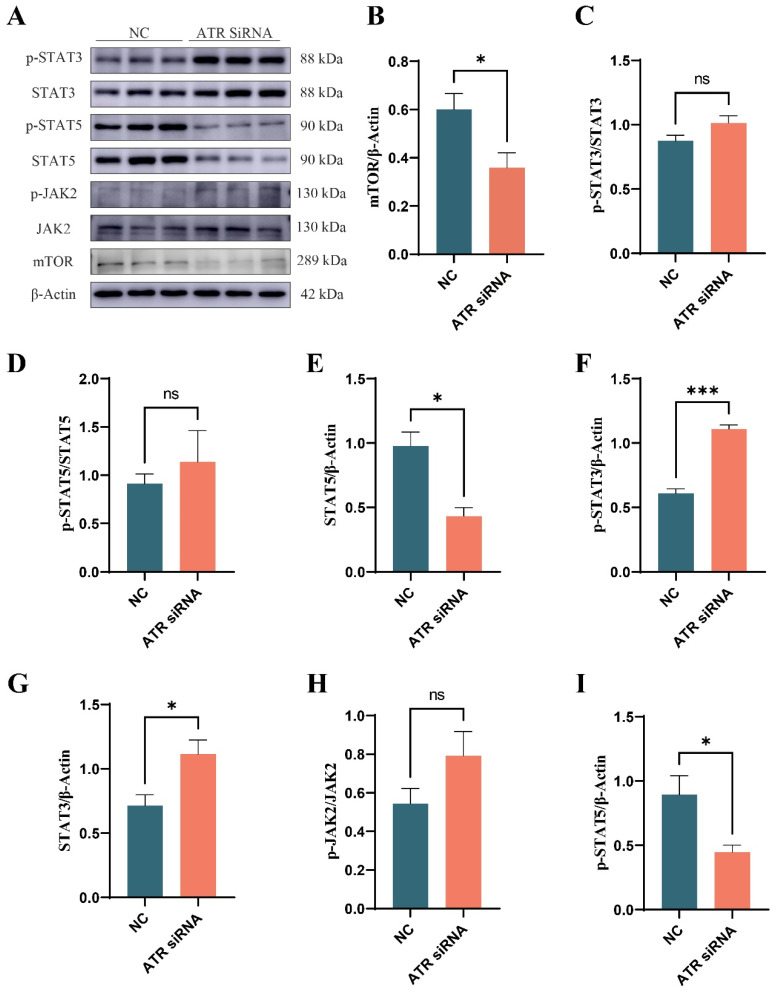
Expression of lactation-related pathway proteins in BMECs after silencing ATR gene. (**A**) Western blot image. (**B**) Grayscale analysis of mTOR protein. (**C**) Grayscale analysis of p-STAT3/STAT3 protein. (**D**) Grayscale analysis of p-STAT5/STAT5 protein. (**E**) Grayscale analysis of STAT5 protein. (**F**) Grayscale analysis of p-STAT3 protein. (**G**) Grayscale analysis of STAT3 protein. (**H**) Grayscale analysis of p-JAK2/JAK2 protein. (**I**) Grayscale analysis of p-STAT5 protein. Significant differences are indicated as * *p* < 0.05, *** *p* < 0.001, ns *p* > 0.05.

The expression levels of p-STAT3/STAT3, p-STAT5/STAT5, p-JAK2/JAK2, and mTOR were examined to evaluate the impact of ATR silencing on lactation-related signaling pathways in BMECs. As shown in Figure 7, the expressions of p-STAT3 (*p* < 0.001) and STAT3 (*p* < 0.05) were increased. However, the ratios of p-STAT3/STAT3, p-STAT5/STAT5, and p-JAK2/JAK2 did not show significant differences (*p* > 0.05). The expressions of mTOR (*p* < 0.05), STAT5 (*p* < 0.01), and p-STAT5 (*p* < 0.05) were significantly decreased.

## 4. Discussion

High-yielding dairy cows with long productive lifespans have remained the goal of the livestock industry. However, intrinsic factors underlying the healthy mammary glands of high-yielding cows remain unclear, and research on how the mammary glands of high- and low-yielding cows change across lactation cycles is lacking. In this study, multi-omics analyses revealed differences in DNA damage and repair between the mammary tissues of high- and low-yielding dairy cows.

Transcriptomic data indicate that upregulated differentially expressed genes (DEGs) are primarily involved in cellular metabolism, protein synthesis, and cell proliferation, suggesting their role in maintaining healthy mammary tissue and facilitating efficient lactation. Conversely, downregulated DEGs are mainly associated with apoptosis, oxidative stress, and DNA damage repair, implying improved regulation in these areas within the mammary tissue of high-yielding cows, thereby reducing cellular damage and preserving function. As parity increases, the gene expression profile in mammary tissue undergoes significant changes. High-parity cows exhibit upregulated genes related to the cell cycle, apoptosis, differentiation, DNA damage response, and the synthesis and secretion of milk components. This phenomenon may be linked to the aging and functional decline of mammary tissue. Therefore, parity significantly influences gene expression and function in mammary tissue.

Notably, the expression of the ATR protein was reduced in the mammary tissues of low-yielding cows, with almost no detectable expression in cows at their sixth lactation. In high-parity cows, the mammary tissue is more susceptible to aging and dysfunction, which negatively impacts milk yield and overall health. ATR is highly expressed in the mammary tissue of high-yielding cows but is minimally expressed in high-parity, low-yielding cows. In vitro qPCR results corroborate this observation. This expression pattern is associated with DEGs enriched in DNA damage repair pathways. The diminished presence of ATR in high-parity cows may exacerbate DNA damage and accelerate the aging of mammary tissue, ultimately reducing milk production. As a crucial DNA damage response (DDR) kinase, ATR is activated in response to replication stress or single-stranded DNA breaks. It initiates cell cycle checkpoints, such as G2/M arrest, to facilitate repair. During lactation, mammary cells undergo extensive replication [9]. Studies have demonstrated that markers of DNA damage in mammary alveolar cells increase during lactation. This physiological DNA damage, associated with replication stress during cell proliferation, activates the DDR, particularly the ATR–CHK1 pathway [5]. This activation triggers the G2/M checkpoint, promoting endoreplication of mammary alveolar cells.

Cellular senescence, a highly complex phenomenon involving multiple physiological processes, refers to the state in which cells enter an irreversible growth arrest [10]. Several factors influence senescence, among which DNA damage is among the central causes [5,11]. ATR is a top-tier kinase in the DNA damage repair pathway within cells. It is responsible for initiating the cellular response to and repair of genomic instability. Once it senses DNA damage and replication stress, ATR is activated rapidly [12]. It participates in cell cycle regulation [12], leading to a decrease in the proportion of cells in the G1 phase and an increase in cells in the G2 and S phases. This occurs because ATR activates cell cycle checkpoints, preventing cells from smoothly entering mitosis. Subsequently [6], cells enter a state of senescence or apoptosis [13]. Moreover, ATR directly phosphorylates over 1000 important substrates within the cell.

The DNA damage repair mechanism is complex and involves numerous proteins. PIG3 (TP53I3) was initially identified as a downstream target gene of p53. PIG3 is involved in activating DNA damage checkpoints, and the absence of endogenous PIG3 sensitizes cells to DNA-damaging agents and impairs DNA repair [14]. PARP1 coalesces with DNA damage sites, preventing the separation of broken DNA ends [15]. The transcription factor Sp1 is associated with DNA repair and senescence. The levels of Sp1 protein decline with age, while its RNA levels remain unchanged, suggesting the involvement of DNA damage signals [16].

In this study, the transcriptomics results showed overexpression of the PIG3 gene in the mammary tissues of high-yielding cows. The increased level of PIG3 protein following ATR silencing in vitro supports the omics findings, indicating that PIG3 may play an important role in ATR-mediated DNA damage repair. After ATR silencing, the level of PARP1 protein increased, while no significant difference was observed for SP1 expression, possibly due to the involvement of different DNA damage repair pathways [17,18].

Apoptosis is a secondary response to DNA damage. In this study, following ATR silencing, the expression of senescence-associated β-galactosidase increased, and that of BAK, cytochrome C, and Cleaved caspase3 was elevated. In contrast, no significant changes were observed in the p-AKT/AKT ratio. After ATR silencing, unrepaired DNA damage may lead to BAK permeabilizing the mitochondrial outer membrane, causing the release of cytochrome C from the mitochondrial intermembrane space. This subsequently activates downstream effector caspases, leading to intrinsic apoptosis.

Senescent cells are typically associated with various pathological characteristics. Jean-Philippe Coppé reported that senescence could promote the malignant transformation of neighboring pre-cancerous cells through the secretion of factors related to inflammation and oncogenes, which he termed “Senescence-Associated Secretory Phenotype (SASP)” [19]. SASP includes pro-inflammatory cytokines (such as IL-1α, IL-1β, IL-6, and IL-8), growth factors (such as HGF, TGF-β, and GM-CSF), chemokines (such as CXCL-1/3 and CXCL-10), and matrix-remodeling enzymes (such as metalloproteinases) [20]. SASP may disrupt tissue homeostasis and induce a pro-inflammatory state. Moreover, DNA damage itself is a potent inflammatory mediator [16,21]. These factors act as a “double-edged sword.” On the one hand, the pro-inflammatory effects of the SASP are part of the body’s normal response, which protects against bacterial and viral infections as well as harmful environmental factors. They play important roles in activating the immune system and promoting the development of mammary cells. However, with increasing age, under the influence of multiple factors, such as declining immune function, autophagy dysregulation, and DNA damage, senescent cells accumulate in large numbers within the body. The SASP can disrupt the microenvironment, damage tissue structure and function, and promote local and systemic age-related pathologies [22]. Therefore, in this study, we assessed SASP and found that the expression levels of IL-6, IL-10, IL-1β, TNF-γ, IL-2, and TNF-α were significantly increased in senescent mammary epithelial cells. However, the lack of significant changes in IL-8 and IL-4 suggests a modular regulation of SASP. IL-8 typically recruits and activates macrophages upon tissue over-injury, induces epithelial–mesenchymal transition, and, combined with an IL-6 to IL-8 ratio of 3.0, upregulates cyclin D1 to drive breast cancer cell proliferation [23]. IL-4 is crucial for macrophage health and aging delay; it activates STAT6 to boost DNA repair gene expression and maintain genomic stability. In STAT6-deficient mice, macrophages show senescence and enhanced SASP [24]. If tissue damage does not strongly activate the pathways triggering IL-8 and IL-4 secretion, their levels likely remain stable.

The decline in the lactation function of mammary epithelial cells is among the manifestations of cellular aging [25]. STAT and mTOR are crucial signaling pathways for mammary lactation and are central to the regulation of lactation. STAT3 and STAT5 are members of the STAT family and are involved in various biological processes, including cell growth and inflammation regulation. STAT3 functions during the involution phase of the bovine mammary gland, where it can induce apoptosis of BMECs and regulate tissue remodeling by upregulating suppressors of cytokine signaling (SOCS) [26]. STAT5, a key molecule in the JAK2–STAT5 signaling pathway, regulates lactation [27]. After calving, BMECs are stimulated by prolactin (PRL), which subsequently activates JAK2. Phosphorylated JAK2 activates downstream STAT5, which then translocates to the nucleus to regulate the transcription of milk protein-related genes [27,28]. mTOR integrates nutritional and hormonal signals, activates downstream ribosomes, promotes milk protein synthesis and lipid production, reprograms mammary cell metabolism, and enhances the expression of lactation-related genes, thereby driving milk secretion [29]. In this study, after ATR silencing, the levels of STAT3 and p-STAT3 increased, while the ratio of p-STAT3/STAT3 remained unchanged. Conversely, the levels of STAT5 and p-STAT5 decreased but the ratio of p-STAT5/STAT5 did not change. This suggests that, in senescent mammary epithelial cells, STAT3 and STAT5 may be involved in related cellular physiological activities, and DNA damage may be involved in a certain regulatory mechanism for the expression of STAT and p-STAT.

In our study, the expression of mTOR protein was reduced in senescent mammary epithelial cells. As an integrator of nutritional and hormonal signals, the mTOR signaling pathway promotes milk protein synthesis by activating ribosomal biogenesis mechanisms and coordinates lipid metabolism pathways to reprogram mammary cell metabolism. This ultimately strengthens the expression profile of lactation-related genes and drives the physiological process of milk secretion [29]. The mTOR signaling cascade is essential for maintaining cellular genomic stability in normal biological processes or during stress responses [30].

## 5. Conclusions

In summary, with the increase in lactation cycles, the expression of ATR protein in the mammary tissues of low-yielding cows decreased, resulting in altered DNA damage repair capacity, the onset of cellular senescence, and suppression of lactation-related signaling pathways.

## Figures and Tables

**Table 1:** Gene sequences.

Target Gene	Forward Primer (5′-3′)	Reverse Primer (5′-3′)
*ATR*	GATAGGTTGTGTTCTGCTAGAGTATGG	TGGCTGGTTGTGCTGGTAGTC
*β-actin*	TCTTCCAGCCTTCCTTCCTG	ACCGTGTTGGCGTAGAGGTC

## Data Availability

The results of differential genes screening are provided in Supplementary File S1.

## Supplementary Materials

The following supporting information can be downloaded at: https://www.mdpi.com/article/10.3390/ani15101419/s1, Table S1: Differentially expressed genes.

## Abbreviations

HP: high-yield parity, LP: low-yield parity, ATR: ataxia telangiectasia and Rad3-related protein, siRNA: small interfering RNA, PBS: phosphate buffer saline, BMECs: bovine mammary epithelial cells, qPCR: quantitative polymerase chain reaction, SDS-PAGE: sodium dodecyl sulfate–polyacrylamide gel electrophoresis, PIG3: p53-inducible gene 3, JAK2: Janus kinase 2, p-JAK2: phospho-Janus kinase 2, SP1: specificity protein 1, STAT3: signal transducer and activator of transcription 3, p-STAT3: phospho-signal transducer and activator of transcription 3, STAT5: signal transducer and activator of transcription 5, p-STAT5: phospho-signal transducer and activator of transcription 5, BAK1: BCL2 antagonist/killer 1, AKT: protein kinase B, mTOR: mammalian target of rapamycin, PARP1: poly ADP-ribose polymerase, ELISA: enzyme-linked immunosorbent assay, IFN-γ: interferon-γ, IL: interleukin, TNF-α: tumor necrosis factor-α, DEGs: differentially expressed genes, FC: fold change, DEPs: differentially expressed proteins, GO: Gene Ontology, KEGG: Kyoto Encyclopedia of Genes and Genomes, SASP: senescence-associated secretory phenotype.
