# Supplementary material for: ATR Deficiency Impairs DNA Damage Repair and Accelerates Cellular Senescence in Bovine Mammary Epithelial Cells, Leading to Lactation Dysfunction
Source: Animals (Basel). 2025 May 14;15(10):1419. doi: 10.3390/ani15101419 (PMC12108212; doi:10.3390/ani15101419)
Supplement: Supplementary file 1 [file animals-15-01419-s001.zip › animals-3539500-supplementary.pdf]

Supplementary File
Table S1

Table S1 Differentially expressed genes

Gene Name	LP5vsHP5		LP6vsHP6	
	log2FoldChange	P value	log2FoldChange	P value
ABHD10	1.120472	0.034108	1.586168837	0.019339338
IL6	1.764549	0.006192	1.452963757	0.044999441
LALBA	1.378848	0.010742	1.72044594	0.047494066
NOP2	-1.04107	0.001556	-1.116812697	0.040232805
SRD5A3	1.485486	0.001313	1.222455936	0.042885139
KLF2	-1.53369	0.00021	-1.532004179	0.017963062
LYRM7	1.513214	0.024714	1.574309606	0.029326775
TP53I3	-1.28204	0.038311	-1.40417355	0.043275242
SLC1A3	1.093871	0.016591	1.427581418	0.006981511
PI16	-1.15311	0.003267	-1.717219331	0.005671331
STX1A	-1.38793	0.045991	-1.718414307	0.031228045
NMUR2	-2.0685	0.006396	2.217991069	0.008528105
CD24	-1.10961	0.031719	1.83945554	0.001120746
ATP6V0D2	1.696723	0.006858	1.95077897	0.00191849
BCL9L	-1.29328	0.002718	-1.252223801	0.017176492
LYPLAL1	1.135089	0.006856	1.10950855	0.047840214
BCAR1	-1.14105	0.016398	-2.267504472	0.001354438
SH2B2	-1.21764	0.012734	-1.516163281	0.030173315
SAP25	-1.01257	0.000579	-1.60881602	0.010023463
SYT7	-1.52586	0.033472	-2.151769727	0.000596909
PRR33	-1.17276	0.02425	-1.562177924	0.02482891
NOL3	1.189388	0.023485	-1.647988242	0.042553493
CYP2S1	1.414594	0.031478	-1.500783861	0.018082618
PCYOX1	1.029264	0.010551	1.732315165	0.029731885
MTRF1	1.002862	0.028975	1.352479379	0.03121134
WDR97	-1.12847	0.019445	-1.994750922	0.004220088
CALB1	4.30955	0.002312	4.519319655	0.000288759
